# Crystal structures of (*S*)-(+)-5-(3-bromo/chloro-4-isopropoxyphen­yl)-5-methyl­imidazolidine-2,4-dione

**DOI:** 10.1107/S2056989015023014

**Published:** 2016-01-16

**Authors:** Shigeru Ohba, Minoru Koura, Hisashi Sumida, Kimiyuki Shibuya

**Affiliations:** a Research and Education Center for Natural Sciences, Keio University, Hiyoshi 4-1-1, Kohoku-ku, Yokohama 223-8521, Japan; b Tokyo New Drug Research Laboratories, Pharmaceutical Division, Kowa Company, Ltd., 2-17-43, Noguchicho, Higashimurayama, Tokyo 189-0022, Japan

**Keywords:** crystal structure, hydantoin, absolute configuration, hydrogen bonding

## Abstract

In (*S*)-(+)-5-(3-bromo-4-isopropoxyphen­yl)-5-methyl­imidazolidine-2,4-dione, C_13_H_15_BrN_2_O_3_, (I), the hydantoin groups are connected *via* inter­molecular N—H⋯O hydrogen bonds, forming a terraced sheet structure. In the chloro analogue, (*S*)-(+)-5-(3-chloro-4-isopropoxyphen­yl)-5-methyl­imidazolidine-2,4-dione, C_13_H_15_ClN_2_O_3_, (II), the inter­molecular N—H⋯O hydrogen-bonding network forms a flat sheet. Comparison of the crystal structures reveals that (II) is more loosely packed than (I).

## Abstract 2

The chiral title compounds are closely related hydantoin derivatives with bromo and chloro substituents at the 3-position of the benzene ring of the isopropoxyphenyl subtituent. In the both crystals, hydantoin groups are connected by N—H⋯O hydrogen bonds, forming two-dimensional sheets, made up from 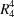
(20) rings.

## Chemical context   

In searching for a new synthetic β-selective agonist toward liver X receptors (LXR), a series of compounds having the hydantoin tail, which may act as a linker, were synthesized and examined (Matsuda *et al.*, 2015 ▸; Koura *et al.*, 2015 ▸). It has been revealed that the chirality of the hydantoin unit is crucial to the LXR activation and β selectivity (Koura *et al.*, 2016 ▸). In the present study, the absolute configuration of the (+)-hydantoin unit, which leads to pharmacological activity, has been determined definitely from anomalous-dispersion effects in diffraction measurements on crystals of the title bromo and chloro derivatives.
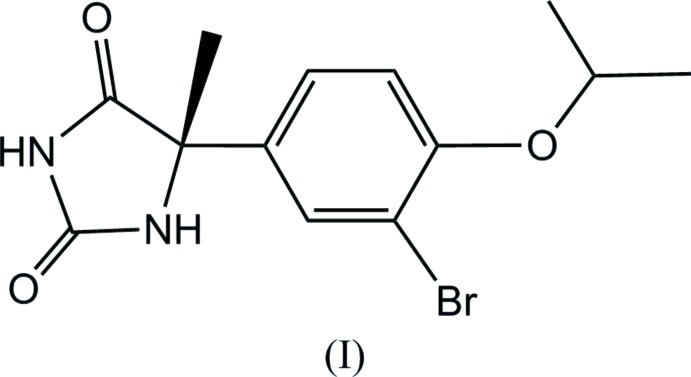
 
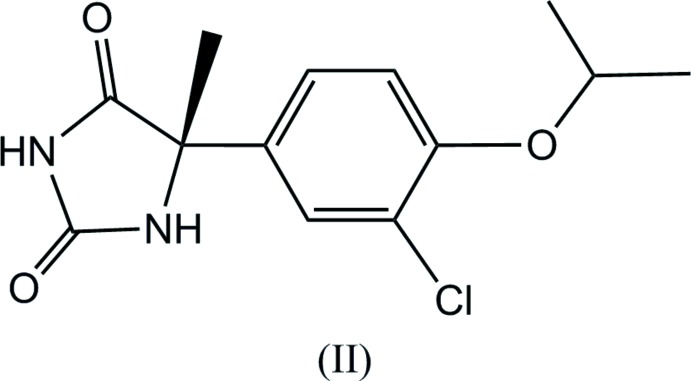


## Structural commentary   

**Figure 1:**
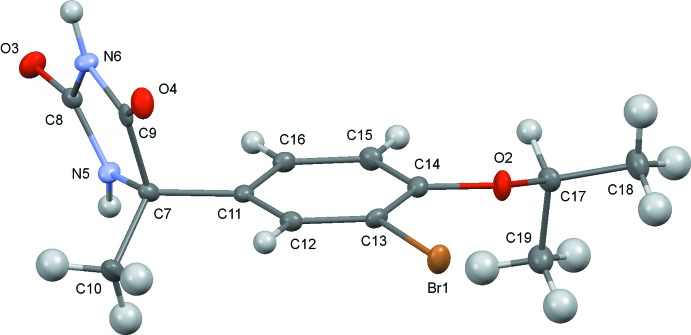
The mol­ecular structure of (I), showing displacement ellipsoids at the 50% probability level.

The conformations of the mol­ecules (I) and (II) are similar to one another (Figs. 1 ▸ and 2 ▸), although the inclination angles of the C11–C16 benzene rings to the hydantoin group around the C7—C11 bond axes differ somewhat, the N5—C7—C11—C16 torsion angles being 12.9 (3)° and −9.8 (2)° for (I) and (II), respectively. The configuration around the asymmetric carbon atom C7 of the (+)-isomer has been determined to *S* for both (I) and (II). It is worthwhile to compare the Flack parameters calculated by classical refinement (Flack, 1983 ▸) and Parsons’ quotient (Parsons *et al.*, 2013 ▸) for these Br and Cl compounds which were measured with Mo *K*α radiation. These values are 0.010 (7) and 0.018 (2) for (I), and 0.010 (50) and 0.009 (8) for (II), respectively. Flack parameters with much smaller s.u. values were obtained by Parsons’ method.

## Supra­molecular features   

**Figure 3:**
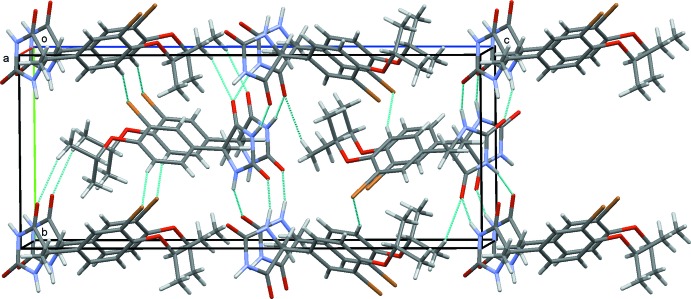
The crystal structure of (I), projected along *a*. Hydrogen bonds are shown as dashed lines.

**Figure 4:**
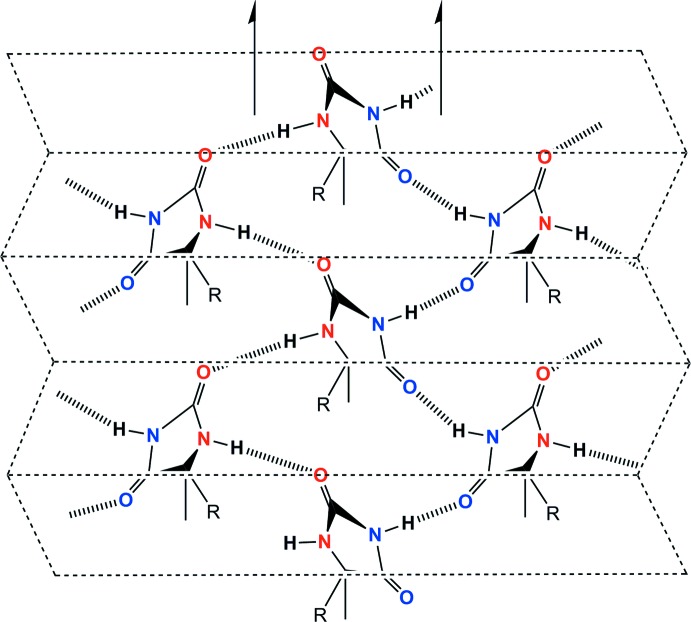
A schematic drawing of the N—H⋯O hydrogen-bonding network in (I). The arrows indicate the twofold screw axes along *a*.

The crystal structure of (I) projected along *a* is shown in Fig. 3 ▸. The hydantoin ring systems are linked by two sets of N—H⋯O hydrogen bonds (Table 1 ▸) and are arranged in zigzag fashion along the twofold screw axes at *z* = 0 and *z* = ½ along *a*. Groups of four mol­ecules are linked by these N—H⋯O hydrogen bonds, generating 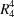
(20) ring motifs, forming terraced sheets parallel to (001) as shown schematically in Fig. 4 ▸. The 3-bromo-4-isopropoxyphenyl groups are accommodated between these sheets and linked by the C—H⋯Br and C—H⋯O hydrogen bonds, forming a three-dimensional architecture.

**Figure 5:**
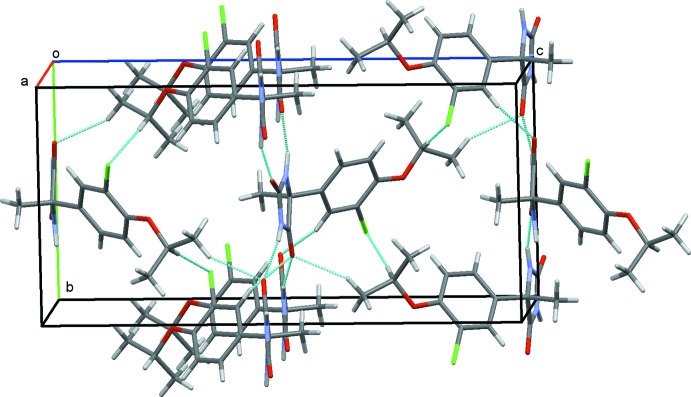
The crystal structure of (II), projected along *a*. Hydrogen bonds are shown as dashed lines.

Both (I) and (II) crystallize in space group *P*2_1_2_1_2_1_ and the lattice constants are roughly similar for both. However, there are both similarities and significant differences in the packing modes between the two closely related mol­ecules. The crystal structure of (II) projected along *a* is shown in Fig. 5 ▸. The hydantoin ring systems again lie approximately on planes at *z* = 0 or *z* = ½, and are connected by N—H⋯O hydrogen bonds (Table 2 ▸), forming a flat sheet parallel to (001). Between these sheets 3-chloro-4-isopropoxyphenyl groups are linked by C—H⋯Cl and C—H⋯O hydrogen bonds, generating a three-dimensional structure of mol­ecules stacked along *a*.

Comparison of the crystal structures reveals that (II) is more loosely packed than (I). There are significant differences in the van der Waals radii of the Br and Cl atoms (1.85 and 1.75 Å, respectively; Bondi, 1964 ▸) which is reflected in the C—*X* bond distances [C13—Br1 = 1.8945 (18) Å in (I); C13—Cl1 1.7396 (16) Å in (II)]. However, the effective volume of the mol­ecule in (II) estimated by *V*/*Z* is larger by *ca* 4% than that for (I). This suggests that the nearly coplanar arrangement of the hydantoin groups in (II) is favorable for the formation of N—H⋯O hydrogen bonds as seen from Table 2 ▸, but it also results in looser mol­ecular packing.

## Database survey   

Structures of 5-phenyl-5-alkyl­hydantoin derivatives have been investigated to review the relationships between the absolute configuration and optical activity. Knabe & Wunn (1980 ▸) determined the absolute configurations of 5,5-disubstituted hydantoins based on their chemical syntheses. According to this assignment, the structure of *S*-(+)-5-phenyl-5-ethyl­hydantoin was reported (Coquerel *et al.*, 1993 ▸). Ferron *et al.* (2006 ▸) determined the configuration of (*R*)-(−)-5-*p*-methyl­phenyl-5-methyl­hydantoin in a chlathrate compound with permethyl­ated β-cyclo­dextrin based on the known absolute configuration of the host. Martin *et al.* (2011 ▸) prepared the diastereomeric salt of (*S*)-(+)-5-phenyl-5-tri­fluoro­methyl­hydantoin with (+)-α-methyl­benzyl­amine to determine the configuration based on the known absolute configuration of the chiral amine. It is noted that the *R* and *S* notation remains unchanged when CH_3_ at the 5-position of the hydantoin is replaced with CF_3_, although the priorities of the substituents in the sequence rule are altered. To our knowledge, the present paper is the first to report the absolute configuration of such compounds determined from anomalous-dispersion effects.

## Synthesis and crystallization   

Compounds (I) and (II) were prepared from the corres­ponding (+)-non-halogeno-derivatives, which were separated from a racemic mixture (Koura *et al.*, 2016 ▸). Prismatic crystals of (I) were grown from ethyl­acetate solution. The specific rotation, [α]_D_, of (I) at 293 K is +79.7° (*c* = 0.98, MeOH, where *c* is the concentration of units gram per 100 cm^−3^).

Plate-like crystals of (II) were grown from ethyl­acetate solution. The specific rotation, [α]_D_, of (II) at 293 K is +81.4° (*c* = 1.0, MeOH).

## Refinement   

Crystal data, data collection and structure refinement details are summarized in Table 3 ▸. All H atoms bound to C and N were positioned geometrically. They were refined as riding, with N—H = 0.88 Å, C—H = 0.95–0.98 Å, and *U*_iso_(H) = 1.2*U*_eq_(C/N) and *U*_iso_(H) = 1.5*U*_eq_(C_methyl_). The thermal displacement ellipsoids of the non-hydrogen atoms of the isoprop­oxy group in (II) are larger than those in (I), suggesting some positional disorder, which was not taken into account in the refinement.

## Supplementary Material

Crystal structure: contains datablock(s) I, II, global. DOI: 10.1107/S2056989015023014/sj5484sup1.cif

Supporting information file. DOI: 10.1107/S2056989015023014/sj5484Isup4.cdx

Supporting information file. DOI: 10.1107/S2056989015023014/sj5484Isup6.cml

Supporting information file. DOI: 10.1107/S2056989015023014/sj5484IIsup5.cdx

Supporting information file. DOI: 10.1107/S2056989015023014/sj5484IIsup7.cml

CCDC references: 1436066, 1439808

Additional supporting information:  crystallographic information; 3D view; checkCIF report

## Figures and Tables

**Figure 2:**
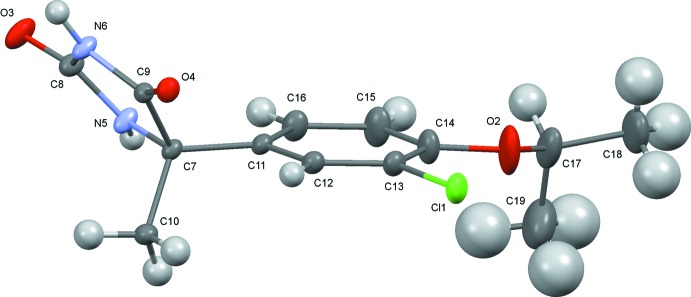
The mol­ecular structure of (II), showing displacement ellipsoids at the 50% probability level.

**Table 1:** Hydrogen-bond geometry (Å, °) for (I)

*D*—H⋯*A*	*D*—H	H⋯*A*	*D*⋯*A*	*D*—H⋯*A*
C18—H18*B*⋯O4^i^	0.98	2.60	3.529 (3)	158
C15—H15⋯Br1^i^	0.95	3.02	3.939 (2)	162
N6—H6⋯O4^ii^	0.88	1.97	2.828 (2)	165
N5—H5⋯O3^iii^	0.88	2.12	2.861 (2)	141

Symmetry codes: (i) 
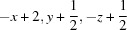
; (ii) 
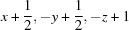
; (iii) 
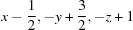
.

**Table 2:** Hydrogen-bond geometry (Å, °) for (II)

*D*—H⋯*A*	*D*—H	H⋯*A*	*D*⋯*A*	*D*—H⋯*A*
N5—H5⋯O3^i^	0.88	2.00	2.8155 (16)	154
N6—H6⋯O4^ii^	0.88	2.03	2.8845 (16)	163
C12—H12⋯O4	0.95	2.57	3.0679 (19)	113
C12—H12⋯O4^iii^	0.95	2.39	3.2294 (18)	147
C17—H17⋯Cl1^iv^	1.00	2.83	3.831 (2)	175
C18—H18*B*⋯O4^iv^	0.98	2.50	3.409 (2)	154

Symmetry codes: (i) 
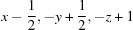
; (ii) 
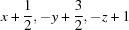
; (iii) 
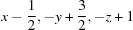
; (iv) 
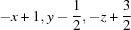
.

**Table 3:** Experimental details

	(I)	(II)
Crystal data		
Chemical formula	C_13_H_15_BrN_2_O_3_	C_13_H_15_ClN_2_O_3_
*M*_r_	327.17	282.72
Crystal system, space group	Orthorhombic, *P*2_1_2_1_2_1_	Orthorhombic, *P*2_1_2_1_2_1_
Temperature (K)	90	90
*a*, *b*, *c* (Å)	6.1840 (3), 9.6495 (4), 23.1111 (10)	7.1397 (3), 10.0128 (4), 20.0431 (8)
*V* (Å^3^)	1379.10 (11)	1432.85 (10)
*Z*	4	4
Radiation type	Mo *K*α	Mo *K*α
μ (mm^−1^)	2.99	0.27
Crystal size (mm)	0.25 × 0.25 × 0.10	0.27 × 0.27 × 0.21

Data collection		
Diffractometer	Bruker D8 VENTURE	Bruker D8 VENTURE
Absorption correction	Integration (*SADABS*; Bruker, 2014 ▸)	Integration (*SADABS*; Bruker, 2014 ▸)
*T*_min_, *T*_max_	0.482, 0.631	0.916, 0.954
No. of measured, independent and observed [*I* > 2σ(*I*)] reflections	31000, 3271, 3206	32943, 3425, 3350
*R*_int_	0.028	0.023
(sin θ/λ)_max_ (Å^−1^)	0.659	0.660

Refinement		
*R*[*F* ^2^ > 2σ(*F* ^2^)], *wR*(*F* ^2^), *S*	0.017, 0.047, 1.29	0.027, 0.079, 1.68
No. of reflections	3271	3425
No. of parameters	175	175
H-atom treatment	H-atom parameters constrained	H-atom parameters constrained
Δρ_max_, Δρ_min_ (e Å^−3^)	0.41, −0.30	0.28, −0.22
Absolute structure	Flack *x* determined using 1301 quotients [(*I* ^+^)−(*I* ^−^)]/[(*I* ^+^)+(*I* ^−^)] (Parsons *et al.*, 2013 ▸).	Flack *x* determined using 1385 quotients [(*I* ^+^)−(*I* ^−^)]/[(*I* ^+^)+(*I* ^−^)] (Parsons *et al.*, 2013 ▸)
Absolute structure parameter	0.018 (2)	0.009 (8)

Computer programs: *APEX2* and *SAINT* (Bruker, 2014 ▸), *SHELXT* (Sheldrick, 2015*a* ▸), *SHELXL2014* (Sheldrick, 2015*b* ▸), *Mercury* (Macrae *et al.*, 2008 ▸) and *publCIF* (Westrip, 2010 ▸).

## supplementary crystallographic information

### Crystal data

**Table:**

C_13_H_15_ClN_2_O_3_	*D*_x_ = 1.311 Mg m^−^^3^
*M**_r_* = 282.72	Melting point = 475–477 K
Orthorhombic, *P*2_1_2_1_2_1_	Mo *K*α radiation, λ = 0.71073 Å
*a* = 7.1397 (3) Å	Cell parameters from 9929 reflections
*b* = 10.0128 (4) Å	θ = 2.9–27.9°
*c* = 20.0431 (8) Å	µ = 0.27 mm^−^^1^
*V* = 1432.85 (10) Å^3^	*T* = 90 K
*Z* = 4	Plate, colorless
*F*(000) = 592	0.27 × 0.27 × 0.21 mm

### Data collection

**Table:**

Bruker D8 VENTURE diffractometer	3350 reflections with *I* > 2σ(*I*)
φ and ω scans	*R*_int_ = 0.023
Absorption correction: integration (*SADABS*; Bruker, 2014)	θ_max_ = 28.0°, θ_min_ = 2.3°
*T*_min_ = 0.916, *T*_max_ = 0.954	*h* = −9→9
32943 measured reflections	*k* = −13→13
3425 independent reflections	*l* = −26→26

### Refinement

**Table:**

Refinement on *F*^2^	Hydrogen site location: inferred from neighbouring sites
Least-squares matrix: full	H-atom parameters constrained
*R*[*F*^2^ > 2σ(*F*^2^)] = 0.027	*w* = 1/[σ^2^(*F*_o_^2^) + (0.0389*P*)^2^] where *P* = (*F*_o_^2^ + 2*F*_c_^2^)/3
*wR*(*F*^2^) = 0.079	(Δ/σ)_max_ = 0.001
*S* = 1.68	Δρ_max_ = 0.28 e Å^−^^3^
3425 reflections	Δρ_min_ = −0.22 e Å^−^^3^
175 parameters	Absolute structure: Flack *x* determined using 1385 quotients [(*I*^+^)-(*I*^-^)]/[(*I*^+^)+(*I*^-^)] (Parsons *et al.*, 2013).
0 restraints	Absolute structure parameter: 0.009 (8)

### Special details

**Table:**

Geometry. All e.s.d.'s (except the e.s.d. in the dihedral angle between two l.s. planes) are estimated using the full covariance matrix. The cell e.s.d.'s are taken into account individually in the estimation of e.s.d.'s in distances, angles and torsion angles; correlations between e.s.d.'s in cell parameters are only used when they are defined by crystal symmetry. An approximate (isotropic) treatment of cell e.s.d.'s is used for estimating e.s.d.'s involving l.s. planes.

### Fractional atomic coordinates and isotropic or equivalent isotropic displacement parameters (Å^2^)

**Table:**

	*x*	*y*	*z*	*U*_iso_*/*U*_eq_	
Cl1	0.27670 (6)	0.69656 (4)	0.65403 (2)	0.02286 (13)	
O2	0.3090 (2)	0.46523 (14)	0.73500 (6)	0.0401 (4)	
O3	1.27973 (17)	0.36910 (11)	0.49720 (6)	0.0240 (3)	
O4	0.88769 (16)	0.72460 (10)	0.52129 (5)	0.0149 (2)	
N5	0.95990 (18)	0.37992 (12)	0.51449 (7)	0.0153 (3)	
H5	0.9349	0.2942	0.5184	0.018*	
N6	1.11959 (17)	0.56832 (12)	0.50854 (6)	0.0149 (3)	
H6	1.2159	0.6228	0.5052	0.018*	
C7	0.81752 (19)	0.48393 (14)	0.51642 (7)	0.0122 (3)	
C8	1.1342 (2)	0.42885 (15)	0.50598 (8)	0.0160 (3)	
C9	0.9406 (2)	0.60975 (14)	0.51672 (7)	0.0121 (3)	
C10	0.7029 (2)	0.48536 (16)	0.45165 (8)	0.0178 (3)	
H10A	0.7874	0.4951	0.4134	0.027*	
H10B	0.6148	0.5604	0.4526	0.027*	
H10C	0.6331	0.4015	0.4476	0.027*	
C11	0.6927 (2)	0.47604 (15)	0.57820 (7)	0.0138 (3)	
C12	0.5631 (2)	0.57769 (15)	0.58963 (7)	0.0154 (3)	
H12	0.5602	0.6531	0.5609	0.018*	
C13	0.4397 (2)	0.56974 (16)	0.64193 (8)	0.0176 (3)	
C14	0.4386 (3)	0.46071 (18)	0.68561 (8)	0.0250 (4)	
C15	0.5703 (3)	0.3610 (2)	0.67485 (9)	0.0308 (4)	
H15	0.5750	0.2864	0.7041	0.037*	
C16	0.6959 (2)	0.36877 (17)	0.62175 (8)	0.0219 (3)	
H16	0.7849	0.2994	0.6153	0.026*	
C17	0.2730 (3)	0.3480 (2)	0.77469 (9)	0.0359 (5)	
H17	0.3949	0.3107	0.7907	0.043*	
C18	0.1633 (3)	0.4001 (3)	0.83411 (10)	0.0452 (6)	
H18A	0.2356	0.4701	0.8565	0.068*	
H18B	0.1397	0.3267	0.8654	0.068*	
H18C	0.0436	0.4370	0.8188	0.068*	
C19	0.1706 (4)	0.2430 (3)	0.73698 (14)	0.0619 (8)	
H19A	0.0587	0.2818	0.7164	0.093*	
H19B	0.1336	0.1712	0.7675	0.093*	
H19C	0.2522	0.2065	0.7021	0.093*	

### Atomic displacement parameters (Å^2^)

**Table:**

	*U*^11^	*U*^22^	*U*^33^	*U*^12^	*U*^13^	*U*^23^
Cl1	0.0193 (2)	0.0269 (2)	0.02235 (19)	0.00684 (16)	0.00608 (15)	−0.00032 (15)
O2	0.0438 (9)	0.0423 (8)	0.0342 (7)	0.0146 (7)	0.0254 (7)	0.0183 (6)
O3	0.0127 (5)	0.0138 (5)	0.0455 (7)	0.0033 (5)	0.0032 (6)	−0.0029 (5)
O4	0.0150 (5)	0.0099 (5)	0.0197 (5)	0.0024 (4)	0.0012 (4)	0.0004 (4)
N5	0.0117 (6)	0.0068 (6)	0.0276 (7)	0.0001 (5)	0.0028 (5)	0.0005 (5)
N6	0.0098 (6)	0.0087 (6)	0.0264 (6)	−0.0009 (5)	0.0009 (5)	−0.0012 (5)
C7	0.0099 (7)	0.0097 (6)	0.0169 (7)	−0.0002 (5)	0.0005 (5)	0.0004 (5)
C8	0.0141 (7)	0.0116 (7)	0.0223 (7)	−0.0008 (6)	−0.0010 (6)	−0.0003 (6)
C9	0.0119 (7)	0.0125 (7)	0.0120 (6)	−0.0020 (5)	0.0002 (5)	0.0008 (5)
C10	0.0146 (7)	0.0214 (8)	0.0175 (7)	−0.0030 (6)	−0.0018 (6)	−0.0007 (6)
C11	0.0106 (7)	0.0153 (7)	0.0156 (6)	−0.0015 (5)	0.0003 (5)	0.0010 (5)
C12	0.0144 (7)	0.0156 (7)	0.0160 (7)	−0.0003 (6)	−0.0005 (6)	0.0031 (6)
C13	0.0141 (7)	0.0192 (7)	0.0195 (7)	0.0036 (6)	0.0008 (6)	0.0007 (6)
C14	0.0263 (9)	0.0285 (9)	0.0201 (8)	0.0049 (8)	0.0087 (7)	0.0088 (7)
C15	0.0338 (10)	0.0295 (9)	0.0292 (9)	0.0090 (9)	0.0097 (8)	0.0167 (8)
C16	0.0213 (8)	0.0206 (8)	0.0238 (8)	0.0050 (7)	0.0021 (7)	0.0076 (6)
C17	0.0293 (10)	0.0476 (11)	0.0307 (9)	0.0059 (9)	0.0117 (9)	0.0219 (9)
C18	0.0439 (13)	0.0628 (15)	0.0288 (10)	0.0004 (11)	0.0148 (9)	0.0171 (10)
C19	0.0543 (17)	0.0669 (17)	0.0646 (16)	−0.0140 (14)	0.0265 (14)	−0.0055 (14)

### Geometric parameters (Å, º)

**Table:**

Cl1—C13	1.7396 (16)	C11—C12	1.395 (2)
O2—C14	1.355 (2)	C12—C13	1.371 (2)
O2—C17	1.441 (2)	C12—H12	0.9500
O3—C8	1.2121 (18)	C13—C14	1.399 (2)
O4—C9	1.2139 (18)	C14—C15	1.389 (3)
N5—C8	1.3479 (19)	C15—C16	1.394 (2)
N5—C7	1.4558 (18)	C15—H15	0.9500
N5—H5	0.8800	C16—H16	0.9500
N6—C9	1.3536 (19)	C17—C19	1.487 (3)
N6—C8	1.4014 (19)	C17—C18	1.518 (3)
N6—H6	0.8800	C17—H17	1.0000
C7—C11	1.5277 (19)	C18—H18A	0.9800
C7—C10	1.535 (2)	C18—H18B	0.9800
C7—C9	1.5360 (19)	C18—H18C	0.9800
C10—H10A	0.9800	C19—H19A	0.9800
C10—H10B	0.9800	C19—H19B	0.9800
C10—H10C	0.9800	C19—H19C	0.9800
C11—C16	1.384 (2)		
			
C14—O2—C17	119.87 (16)	C12—C13—C14	121.82 (15)
C8—N5—C7	112.83 (12)	C12—C13—Cl1	119.59 (12)
C8—N5—H5	123.6	C14—C13—Cl1	118.57 (12)
C7—N5—H5	123.6	O2—C14—C15	126.84 (16)
C9—N6—C8	112.33 (13)	O2—C14—C13	115.78 (16)
C9—N6—H6	123.8	C15—C14—C13	117.37 (15)
C8—N6—H6	123.8	C14—C15—C16	120.93 (16)
N5—C7—C11	113.08 (12)	C14—C15—H15	119.5
N5—C7—C10	110.89 (12)	C16—C15—H15	119.5
C11—C7—C10	112.02 (12)	C11—C16—C15	120.96 (16)
N5—C7—C9	100.80 (11)	C11—C16—H16	119.5
C11—C7—C9	111.90 (11)	C15—C16—H16	119.5
C10—C7—C9	107.50 (12)	O2—C17—C19	112.53 (18)
O3—C8—N5	129.09 (13)	O2—C17—C18	104.23 (17)
O3—C8—N6	124.11 (14)	C19—C17—C18	112.8 (2)
N5—C8—N6	106.80 (13)	O2—C17—H17	109.0
O4—C9—N6	126.38 (14)	C19—C17—H17	109.0
O4—C9—C7	126.82 (14)	C18—C17—H17	109.0
N6—C9—C7	106.75 (12)	C17—C18—H18A	109.5
C7—C10—H10A	109.5	C17—C18—H18B	109.5
C7—C10—H10B	109.5	H18A—C18—H18B	109.5
H10A—C10—H10B	109.5	C17—C18—H18C	109.5
C7—C10—H10C	109.5	H18A—C18—H18C	109.5
H10A—C10—H10C	109.5	H18B—C18—H18C	109.5
H10B—C10—H10C	109.5	C17—C19—H19A	109.5
C16—C11—C12	118.27 (14)	C17—C19—H19B	109.5
C16—C11—C7	122.80 (14)	H19A—C19—H19B	109.5
C12—C11—C7	118.85 (12)	C17—C19—H19C	109.5
C13—C12—C11	120.62 (13)	H19A—C19—H19C	109.5
C13—C12—H12	119.7	H19B—C19—H19C	109.5
C11—C12—H12	119.7		
			
C8—N5—C7—C11	−126.78 (14)	C10—C7—C11—C12	−60.26 (17)
C8—N5—C7—C10	106.43 (14)	C9—C7—C11—C12	60.55 (17)
C8—N5—C7—C9	−7.18 (15)	C16—C11—C12—C13	−1.3 (2)
C7—N5—C8—O3	−173.72 (17)	C7—C11—C12—C13	175.49 (14)
C7—N5—C8—N6	5.83 (18)	C11—C12—C13—C14	0.0 (2)
C9—N6—C8—O3	178.00 (15)	C11—C12—C13—Cl1	−178.80 (11)
C9—N6—C8—N5	−1.57 (19)	C17—O2—C14—C15	−12.6 (3)
C8—N6—C9—O4	179.17 (14)	C17—O2—C14—C13	168.51 (17)
C8—N6—C9—C7	−2.93 (18)	C12—C13—C14—O2	−179.76 (16)
N5—C7—C9—O4	−176.28 (14)	Cl1—C13—C14—O2	−0.9 (2)
C11—C7—C9—O4	−55.83 (19)	C12—C13—C14—C15	1.2 (3)
C10—C7—C9—O4	67.57 (18)	Cl1—C13—C14—C15	−179.94 (15)
N5—C7—C9—N6	5.83 (15)	O2—C14—C15—C16	179.90 (18)
C11—C7—C9—N6	126.28 (13)	C13—C14—C15—C16	−1.2 (3)
C10—C7—C9—N6	−110.32 (13)	C12—C11—C16—C15	1.3 (2)
N5—C7—C11—C16	−9.8 (2)	C7—C11—C16—C15	−175.34 (16)
C10—C7—C11—C16	116.38 (16)	C14—C15—C16—C11	0.0 (3)
C9—C7—C11—C16	−122.81 (15)	C14—O2—C17—C19	−71.8 (2)
N5—C7—C11—C12	173.55 (13)	C14—O2—C17—C18	165.63 (17)

### Hydrogen-bond geometry (Å, º)

**Table:**

*D*—H···*A*	*D*—H	H···*A*	*D*···*A*	*D*—H···*A*
N5—H5···O3^i^	0.88	2.00	2.8155 (16)	154
N6—H6···O4^ii^	0.88	2.03	2.8845 (16)	163
C12—H12···O4	0.95	2.57	3.0679 (19)	113
C12—H12···O4^iii^	0.95	2.39	3.2294 (18)	147
C17—H17···Cl1^iv^	1.00	2.83	3.831 (2)	175
C18—H18*B*···O4^iv^	0.98	2.50	3.409 (2)	154

Symmetry codes: (i) *x*−1/2, −*y*+1/2, −*z*+1; (ii) *x*+1/2, −*y*+3/2, −*z*+1; (iii) *x*−1/2, −*y*+3/2, −*z*+1; (iv) −*x*+1, *y*−1/2, −*z*+3/2.
